# Utilization of remote monitoring among patients receiving cardiac resynchronization therapy and comparison between Asia and the Americas

**DOI:** 10.1016/j.hroo.2022.06.013

**Published:** 2022-12-16

**Authors:** Niraj Varma, Yusuke Kondo, Seung-Jung Park, Angelo Auricchio, Michael R. Gold, John Boehmer, Ulhas Pandurangi, Eiichi Watanabe, Kwangdeok Lee, Jagmeet P. Singh

**Affiliations:** ∗ Cleveland Clinic London, London, United Kingdom; † Chiba University Graduate School of Medicine, Chiba, Japan; ‡ Samsung Medical Center, Seoul, Republic of Korea; § Cardiocentro Ticino, Lugano, Switzerland; ‖ Medical University of South Carolina, Charleston, South Carolina; ¶ Pennsylvania State University, Hershey, Pennsylvania; # Madras Medical Mission, Chennai, India; ∗∗ Fujita Health University Bantane Hospital, Nagoya, Japan; †† Abbott, Plano, Texas; ‡‡ Massachusetts General Hospital, Boston, Massachusetts

**Keywords:** Asia, Remote monitoring, CRT, Nonresponders, Disparities

**Key Findings**

▪ Digital health technologies have been advocated as instruments to resolve worldwide disparities in health care. Among these, remote monitoring of cardiac implantable electronic devices is recommended as standard of care and may be particularly useful for more complex devices and sicker patients. However, international adoption has not been characterized.
▪ Among patients receiving cardiac resynchronization therapy (CRT), devices implanted in the Americas had remote monitoring implemented in approximately 60%, contrasting with only <6% in Asia. Following diagnosis of nonresponse, there was no change in remote monitoring utilization.
▪ Barriers to remote monitoring need to be identified to improve patient care of patients receiving CRT. This is important to this high-risk group of patients and to adoption of digital health technologies in general.

There is increasing momentum in application of digital solutions in medicine to improve patient outcomes and reduce global health inequities.^1^^,^^2^ Remote monitoring (RM) of patients with cardiac implantable electronic devices received a Class 1 recommendation in 2015 in the United States.^3^ However, adoption varies by device type (eg, pacemakers less frequently) and patient condition, possibly because sicker patients and/or those with more complex devices are perceived to have the most to gain. Cardiac resynchronization therapy (CRT) is the most complex cardiac implantable electronic device, and “nonresponders” (CRT-NR) have one of the poorest prognoses among heart failure patients.^4^ RM enables early detection of potential precipitants of decompensation (eg, atrial fibrillation, loss of %CRT pacing, volume changes), and thereby facilitates early preemptive intervention to improve patient outcomes.^5^ Nevertheless, utilization among CRT patients is not well characterized. Moreover, little is known of practice in Asia.

We contrasted RM use among CRT recipients in Asia vs the United States, before and after the determination of “nonresponse” status, in the international, multicenter, prospective ADVANCE CRT registry, which enrolled the largest studied cohort of Asian CRT patients in global trials.

ADVANCE CRT was a prospective parallel cohort study of CRT follow-up in the Americas vs Asia. Overall results were reported previously.^4^ In brief, during 2013–2015, the study enrolled patients receiving Abbott CRT implants for standard indications. The registry was approved by the institutional review board at each participating site, and all patients provided written informed consent before enrollment. Sites followed each patient every 3 months for 1 year. RM was advised but not mandated. Response status was evaluated using the Clinical Composite Score 6 months postimplant. Subsequent treatment strategies were assessed, including the use of RM (prespecified analysis). Practice was compared between Asia and the Americas.

More patients were enrolled in the Americas (total 653 [United States 604, Brazil 23, Colombia 23, Argentina 5]) than in Asia (total 231 [India 156, China 30, Japan 25, South Korea 20]). From implant to 6 months in Asia, 94.4% of patients were followed with in-clinic visits only (Figure 1). RM (with or without an in-clinic visit) was used in 5.6% of patients. Among American patients, RM was used in the majority (58.5%). More Asian patients responded to CRT (85.7% [198/231] vs 67.5% [441/653] Americans, *P* < .001).

**Figure 1:**
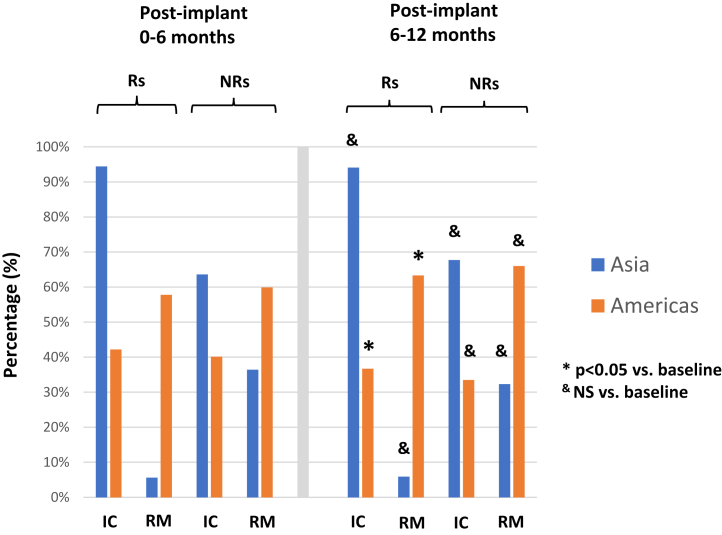
Contrasting remote monitoring utilization between cardiac resynchronization therapy responders (R) and nonresponders (NR) in Asia vs Americas. IC = in-clinic; RM = remote monitoring.

Following assessment of response, in the Americas RM significantly increased among CRT responders (57.8% [255/441] to 63.3% [269/425], *P* = .029) and trended upwards in the CRT-NR cohort (59.9% [127/212] vs 66% [134/200], *P* = .208). However, among Asians, RM use did not change in the CRT responder or CRT-NR group (pre vs post 5.6% [11/198] vs 5.9% [10/170], *P* = .317; 36.4% [12/33] vs 32.3% [10/31], respectively, *P* = .157).

This study informs on practice patterns of RM utilization among CRT recipients internationally. Utilization was negligible in Asia, where in-person follow-up was strongly preferred. Although 10-fold higher in the Americas, level was still less than two-thirds of patients, despite recommendations. Of most concern, RM use did not increase significantly following the determination of high-risk CRT-NR in either the Americas or Asia.

The reasons for the lack of adoption of RM in Asia were not identified in this study. Possibly, the cost of RM-capable devices, increased service burden associated with specialized staffing, lack of reimbursement, lack of physician awareness, and/or the need for more evidence for improved clinical outcome, as well as the “digital divide,” may all inhibit RM adoption.

## Limitations

CRT-NRs numbered very few in Asia. Practice post-COVID may differ and it is necessary to conduct a detailed survey for each country (noting RM received a Class IA recommendation in Japan in 2021).

## Conclusion

Adoption of RM is relatively minor in Asia, even when encouraged and under trial conditions. Barriers need to be identified and resolved to enable the application of digital health care worldwide as advocated by the World Health Organization.^1^^,^^2^
